# Supplementary material for: FastqPuri: high-performance preprocessing of RNA-seq data
Source: BMC Bioinformatics. 2019 May 3;20:226. doi: 10.1186/s12859-019-2799-0 (PMC6500068; doi:10.1186/s12859-019-2799-0)
Supplement: Supplementary file 2 — Archive of FastqPuri. Archive containing all files needed to install and run FastqPuri v1.0.6. Date stamp March 22, 2019. (GZ 47,819 kb) [file 12859_2019_2799_MOESM2_ESM.gz › FastqPuri-1.0.6/html/struct__stats__TF.html]

FastqPuri: \_stats\_TF Struct Reference


FastqPuri

Public Attributes |
List of all members

\_stats\_TF Struct Reference

collects stats info from the filtering procedure
More...

`#include <io_trimFilter.h>`

|  |  |
| --- | --- |
| Public Attributes | |
| int | filters [NFILTERS] |
|  | |
| int | trimmed [NFILTERS] |
|  | |
| int | discarded [NFILTERS] |
|  | |
| int | good |
|  | |
| int | nreads |
|  | |

## Detailed Description

collects stats info from the filtering procedure

## Member Data Documentation

## ◆ discarded

int \_stats\_TF::discarded[NFILTERS]

# discarded reads: ADAP, CONT, LOWQ, NNNN

## ◆ filters

int \_stats\_TF::filters[NFILTERS]

Using filters for: ADAP, CONT, LOWQ, NNNN

## ◆ good

int \_stats\_TF::good

# good reads

## ◆ nreads

int \_stats\_TF::nreads

total number of reads in the fq file

## ◆ trimmed

int \_stats\_TF::trimmed[NFILTERS]

# trimmed reads by: ADAP, CONT, LOWQ, NNNN

---

The documentation for this struct was generated from the following file:

- include/io\_trimFilter.h


---

Generated on Mon Mar 19 2018 23:42:01 for FastqPuri by  

 1.8.14
